# Quantitative Proteomic Profiling Identifies a Potential Novel Chaperone Marker in Resistant Breast Cancer

**DOI:** 10.3389/fonc.2021.540134

**Published:** 2021-02-25

**Authors:** Karen M. Gallegos, Jankiben R. Patel, Shawn D. Llopis, Rashidra R. Walker, A. Michael Davidson, Wensheng Zhang, Kun Zhang, Syreeta L. Tilghman

**Affiliations:** ^1^ Division of Pharmaceutical Sciences, College of Pharmacy and Pharmaceutical Sciences, Florida A&M University, Tallahassee, FL, United States; ^2^ Division of Basic Pharmaceutical Sciences, College of Pharmacy, Xavier University of Louisiana, New Orleans, LA, United States; ^3^ Division of Mathematical and Physical Sciences, Department of Computer Science, College of Arts and Sciences, Xavier University of Louisiana, New Orleans, LA, United States

**Keywords:** aromatase inhibitors, breast cancer, cancer stem cells, letrozole resistance, translation, midasin

## Abstract

Development of aromatase inhibitor resistant breast cancer among postmenopausal women continues to be a major clinical obstacle. Previously, our group demonstrated that as breast cancer cells transition from hormone-dependent to hormone-independent, they are associated with increased growth factor signaling, enhanced cellular motility, and the epithelial to mesenchymal transition (EMT). Given the complexity of cancer stem cells (CSC) and their implications on endocrine resistance and EMT, we sought to understand their contribution towards the development of aromatase inhibitor resistant breast cancer. Cells cultured three dimensionally as mammospheres are enriched for CSCs and more accurately recapitulates tumors *in vivo*. Therefore, a global proteomic analysis was conducted using letrozole resistant breast cancer cells (LTLT-Ca) mammospheres and compared to their adherent counterparts. Results demonstrated over 1000 proteins with quantitative abundance ratios were identified. Among the quantified proteins, 359 were significantly altered (*p* < 0.05), where 173 were upregulated and 186 downregulated (*p* < 0.05, fold change >1.20). Notably, midasin, a chaperone protein required for maturation and nuclear export of the pre-60S ribosome was increased 35-fold. Protein expression analyses confirmed midasin is ubiquitously expressed in normal tissue but is overexpressed in lobular and ductal breast carcinoma tissue as well as ER+ and ER- breast cancer cell lines. Functional enrichment analyses indicated that 19 gene ontology terms and one KEGG pathway were over-represented by the down-regulated proteins and both were associated with protein synthesis. Increased midasin was strongly correlated with decreased relapse free survival in hormone independent breast cancer. For the first time, we characterized the global proteomic signature of CSC-enriched letrozole-resistant cells associated with protein synthesis, which may implicate a role for midasin in endocrine resistance.

## Introduction

Aromatase inhibitors (AI), e.g., letrozole, are the first-line treatment for estrogen receptor positive (ER+) breast cancer in post-menopausal women. Despite widespread successful usage of letrozole, resistance to therapy, tumor relapse, and metastasis remain the principle causes of death for breast cancer patients (1, 2). While there are no cures for AI-resistant breast cancer, previous reports demonstrate AI resistance is associated with increased growth factor signaling (i.e., HER2) (3–5). In addition, we previously identified a global proteomic signature of letrozole resistance that was associated with enhanced cellular motility, estrogen independence (6) and the epithelial to mesenchymal transition (EMT) (7). Although the mortality rates for ER+ breast cancer in the US have declined due to successful endocrine therapy (AI and selective estrogen receptor modulators, SERMS), the development of resistance is still a critical lingering problem. In order to develop improved therapies for highly metastatic breast tumors, it is crucial to uncover the molecular underpinnings that drive resistance and proliferation. An exacerbating factor is the emergence of a small sub-population of breast cancer stem cells (CSC) in AI-resistant breast cancer (8). This is a rate-limiting factor as these pluripotent cells are *de novo* resistant to radiation and chemotherapy. While conventional therapeutic approaches decrease the tumor burden, those approaches are unable to target the CSC population, which usually drives tumor progression, invasion, and metastatic disease (9). To gain an insight of those molecular mechanisms, we identified a chaperone, midasin, as a potential marker for AI-resistant cancer in CSC-enriched mammospheres.

Malignant cancer cells including the CSC population proliferate continuously, thereby requiring enhanced translation including increased ribosome subunit synthesis; hence much of the cell energy is directed to the protein synthesis machinery (10). Ribosomes are the fundamental macromolecular machines at the core of translation. Formation of the 40S and 60S pre-ribosomal subunits are catalyzed by approximately 200 biogenesis factors that participate in the successive assembly and maturation steps, eventually leading to mature 40S and 60S ribosomal subunits  (11–14). Among these are several energy-consuming enzymes including the midasin chaperone. (15–18). A homolog of midasin, Rea1, was initially identified in yeast (19, 20). It comprises an N-terminal domain, followed by six **A**TPase **a**ssociated with diverse cellular **a**ctivities (AAA) regions forming a ring, a linker domain, an acidic domain containing 35–40% aspartate and glutamate, and a carboxy-terminal M-domain that possesses Metal Ion Dependent Adhesion Site (MIDAS) sequence motifs and is homologous to the I-domain of integrins (15). Translation is a fine-tuned and tightly regulated process contributing to normal cell growth and development. It is well known that cancer cells have an increased demand for protein synthesis that is accomplished through aberrant changes in ribosome biogenesis (21). Likewise, inappropriate protein synthesis is associated with cancer. (22) Additionally, deregulation of protein synthesis could enable cells to acquire the classic hallmarks of cancer including, evading growth suppressors, sustaining proliferative signaling, activation of invasion and metastasis, and resisting cell death (23, 24). Although progress has been made towards demonstrating that changes in the quality and quantity of ribosomes can alter translation (25, 26), therapeutically targeting these ribosomal alterations has been unsuccessful.

Currently, effective targeted approaches to combat endocrine resistant breast cancer as well as other chemotherapy refractory cancers are lacking, in part due to an inability to inhibit CSCs and completely unravel the rate-limiting proteins and pathways that drive metastatic disease. To understand the contribution of CSCs on AI-resistance, we perform a quantitative proteomic analysis comparing letrozole-resistant breast cancer cells (LTLT-Ca) cultured two-dimensionally (2D) versus three-dimensionally (3D) as CSC-enriched mammospheres. We chose to use a 3D model system as 2D cultures do not represent the native events of breast cancer progression. Three-dimensional culture systems are advantageous because cells can be cultured in a spatially relevant manner that encourages cell and cell-matrix interactions that closely mimic the tumor environment and acquire morphological and cellular characteristics relevant to those of tumors *in vivo* (27). Since our previous study compared the proteomic profile of letrozole-sensitive vs letrozole-resistant cells cultured 2D, we were interested in capturing how the use of a more physiologically relevant model system would impact the proteome. Therefore, the study objective was to identify novel candidate proteins involved in endocrine resistance within the letrozole-resistant mammosphere cell population compared to cells cultured 2D.

## Materials and Methods

### Cell Culture

Human LTLT-Ca cells are long-term letrozole treated MCF-7 cells and human AC-1 breast cancer cells are letrozole-sensitive MCF-7 cells. AC-1 cells were stably transfected with the human aromatase gene and the LTLT-Ca cells were derived from the AC-1 cells. Both were generously provided by the late Dr. Angela Brodie at University of Maryland. LTLT-Ca cells were cultured in 75-cm^2^ flasks in phenol red-free IMEM (Invitrogen) supplemented with 10% charcoal-stripped fetal bovine serum (CS-FBS), 100 U/ml penicillin G sodium, 100 µg/ml streptomycin sulfate, and 0.25 µg/ml amphotericin B, 750 µg/ml geneticin (Invitrogen), and 1 µM letrozole (Sigma). AC-1 cells were cultured in 5% fetal bovine serum (FBS), 100 U/ml penicillin G sodium, 100 µg/ml streptomycin sulfate, and 0.25 µg/ml amphotericin B, and 750 µg/ml geneticin (Invitrogen). The culture flasks were maintained in a humidified atmosphere of 5% CO_2_ at 37°C. The LTLT-Ca cells were isolated from tumors of aromatase transfected MCF-7 cells grown in ovariectomized severe combined immunodeficiency (SCID) mice following 56 weeks of treatment with letrozole as previously described (28). After long-term letrozole treatment, the tumors acquired the ability to proliferate in the presence of the drug. Mycoplasma testing has been performed on all cell lines. T47D letrozole-sensitive (T47Darom) and T47D letrozole-resistant (T47DaromLR) cells were cultured as previously described by Gupta et al., (29). The MDA-MB-231 cell line (human breast cancer cells negative for ER, PR and Her2/neu) were acquired from the American Type Culture Collection (Manassas, VA, USA) and cultured as previously described (30). All cell lines were authenticated using Short Tandem Repeat (STR) analysis as described in 2012 in ANSI Standard (ASN-0002) by the ATCC Standards Development Organization and by Dr. L. Kerrigan’s group (31). In brief, 17 STR loci plus the gender determining locus, Amelogenin, were amplified using the commercially available PowerPlex^®^ 18D Kit (Promega). The cell line samples were processed using the ABI Prism^®^ 3500 xL Genetic Analyzer. Data were analyzed using GeneMapper^®^ ID-X v1.2 software (Applied Biosystems). The authentication results verified that AC-1 and LTLT-Ca cell lines, T47D variant cell lines, and the MDA-MB-231 cell line shared greater than 85% homology with the MCF-7 cell line, T47D cell line, and the MDA-MB-231 cell line, respectively. Cell lines with ^®^ 80% match are considered to be related and derived from a common ancestry.

### Mammosphere Formation Assay

LTLT-Ca cells were grown to 80–90% confluence and after media was removed, cells were rinsed twice with Hank’s Balanced Salt Solution (HBSS) to remove residual culture media. Cells were gently scraped and resuspended in 10 ml of MammoCult™ media (Stemcell Technologies). Afterwards, cells were centrifuged at 500 g for 3 min at room temperature. The supernatant was discarded and the pellet was resuspended into a single cell suspension in 2 ml of MammoCult™ media. Cell concentration and viability was determined with Trypan Blue exclusion. Cells were enumerated and 100,000 cells were seeded in ultra-low adherent plates. The cultures were incubated in a 5% CO_2_, humidified incubator at 37° C for 7 days and spheres greater than 60 µm were counted and recorded. Mammosphere formation was identified by light microscopy and then harvested as indicated in western blot analysis.

### Western Blotting Analysis

2D and 3D cultured cells were homogenized in cold RIPA buffer supplemented with 2x protease and phosphatase inhibitors (ThermoFisher Scientific). The supernatant was incubated with Laemmli protein sample buffer (Bio-Rad) at 70°C for 10 min. About 75 µg of denatured protein was separated on 7.5% Mini-PROTEAN^®^ TGX™ Precast Protein Gels (Bio-Rad) and transferred to PVDF membranes. All blots were blocked for 1 h with 5% Bovine Serum Albumin (BSA) in Phosphate-Buffered Saline, 0.1% Tween (PBS-T) buffer. Following incubation with anti-midasin antibody (Sigma Aldrich) and secondary antibodies, the blots were visualized using ChemiDoc XRS imaging system (BioRad) and detected with the Clarity Max Western ECL Substrate (BioRad). The exposure time was automatically detected by the imaging system. The protein bands were analyzed using Image Lab software (BioRad). Arbitrary densitometry units were quantified and expressed as mean ± standard deviation. Midasin protein expression was normalized to housekeeping protein bands (GAPDH). All experiments were performed with *n* ≥ 3 and a total of 3 biological replicates were performed.

### Immunohistochemistry

The immunostaining protocol for ICC can be found on the open access repository for science methods at: https://www.protocols.io/view/hpa-cell-atlas-standard-immunostaining-protocol-x2dfqa6.

To provide an overview of protein expression patterns, all images of tissues stained by immunohistochemistry were manually annotated by a specialist followed by verification by a second specialist. Annotation of each different normal and cancer tissue is performed using fixed guidelines for classification of immunohistochemical results. Each tissue is examined for representability, and subsequently immunoreactivity in the different cell types present in normal or cancer tissues was annotated. Basic annotation parameters include an evaluation of i) staining intensity (negative, weak, moderate or strong), ii) fraction of stained cells (<25%, 25%–75% or >75%) and iii) subcellular localization (nuclear and/or cytoplasmic/membranous). The scanned images were viewed in 20x magnification in the freely available database (www.proteinatlas.org). If there was any discordance between the observers, a shared review of the images was performed to obtain a common interpretation.

### Cell Lysis for Proteomic Analysis

LTLT-Ca cells were cultured to 80% confluence in the medium as described above, and washed with cold HBSS three times, then collected with cell scraper. NP40 cell lysis buffer (Invitrogen) containing additional 1 mM of phenylmethylsulfonyl fluoride (PMSF) and protease inhibitor cocktail (Sigma) was used to extract total cellular proteins. The concentration of proteins was measured with BCA assay (Pierce Biotechnology, Rockford, IL). The cell lysates were stored at -80 °C before further processing. There was a total of 3 biological replicates for each sample.

### Trypsin Digestion for Proteomic Analysis

Protein samples were digested with sequencing grade modified trypsin (Promega Corp) according to manufacturer’s instructions. Briefly, 45 μl of 200 mM triethyl ammonium bicarbonate (TEAB) was added to aliquots of 100 μg of protein sample and the final volume was adjusted to 100 μl with ultrapure water. A total of 5 μl of 200 mM Tris (2-carboxyethyl) phosphine (TCEP) was added and the resulting mixture was incubated for 1 h. Following the addition of 5 μl of 375 mM iodoacetamide, the mixture was incubated for 30 min in the dark. After incubation, 1 ml of pre-chilled acetone was added and the precipitation was allowed to proceed overnight. The acetone-precipitated protein pellets were suspended with 100 μl of 200 mM TEAB and 2.5 μg of trypsin was added to digest the sample overnight at 37° C.

### Tandem Mass Tags (TMT) Labeling for Proteomic Analysis

Tandem mass tags TMT6 (Thermo Scientific) with different molecular weights (126 ~ 131 Da) were applied as isobaric tags for relative and absolute quantification during mass spectrometry analysis. According to the manufacturer’s protocols, the digested samples were individually labeled with TMT6 reagents for 1h as follows: three 100-µg aliquots of digested peptides from LTLT-Ca adherent cells were each labeled with a different isobaric tag (TMT126, 127, and 128, respectively). Likewise, 100-µg aliquots of peptides from LTLT-Ca mammospheres were labeled with TMT129, 130, and 131 mass tags, respectively. The labeling reaction was quenched with 5% hydroxylamine. Finally, the six labeled peptide aliquots were combined for subsequent fractionation.

### Fractionation of Labeled Peptide Mixture Using a Strong Cation Exchange Column

The combined TMT labeled peptide mixture was fractionated with a strong cation exchange column (SCX) (Thermo Scientific) on a Shimadzu 2010 HPLC equipped with a UV detector (Shimadzu). Mobile phase consisted of buffer A (5 mM KH_2_PO_4_, 25% acetonitrile, pH 2.8) and buffer B (buffer A plus 350 mM KCl). The column was equilibrated with Buffer A for 30 min before sample injection. The mobile phase gradient was set as follows at a flow rate of 1.0 ml/min: (a) 0 to 10 min: 0% buffer B; (b) 10 to 40 min: 0% to 25% Buffer B, (c) 40 to 45 min: 25% to 100% Buffer B; (d) 45 to 50 min: 100% buffer B; (e) 50 to 60 min: 100% to 0% buffer B; (f) 60 min to 90 min: 0% buffer B. A total of 60 fractions were initially collected, combined and lyophilized into 15 final fractions based on SCX chromatographic peaks.

### Desalination of Fractionated Samples for Proteomic Analysis

A C_18_ solid-phase extraction (SPE) column (Hyper-Sep SPE Columns, Thermo-Fisher Scientific) was used to desalt all collected fractions. The combined 15 fractions were each adjusted to a final volume of 1 ml containing 0.25% (v/v) trifluoroacetic acid (TFA, Sigma). The C_18_ SPE columns were conditioned before use by filling them with 1 ml acetonitrile and allowing the solvent to pass through the column slowly. The columns were then rinsed three times with 1 ml 0.25% TFA solution. The fractions were loaded on top of the SPE cartridge and allowed to elute slowly. Columns were washed four times with 1 ml 0.25% TFA aliquots before the peptides were eluted with 3x 400 µl of 80% acetonitrile/0.1% formic acid.

### Liquid Chromatography Linked to Tandem Mass Spectrometry (LC–MS/MS) Analysis on LTQ-Orbitrap

Peptides were analyzed on an LTQ-Orbitrap XL instrument (Thermo-Fisher Scientific) coupled to an Ultimate 3000 Dionex nanoflow LC system (Dionex). High mass resolution was used for peptide identification and high energy collision dissociation (HCD) was employed for reporter ion quantification. The RP-LC system consisted of a peptide Cap-Trap cartridge (0.5 x 2 mm) (Michrom BioResources) and a prepacked BioBasic C_18_ PicoFrit analytical column of 75 μm i.d. × 15 cm length (New Objective) fitted with a FortisTip emitter tip. Samples were loaded onto the trap cartridge and washed with mobile phase A (98% H_2_O, 2% acetonitrile and 0.1% formic acid) for concentration and desalting. Subsequently, peptides were eluted over 180 min from the analytical column *via* the trap cartridge using a linear gradient of 6%–100% mobile phase B (20% H_2_O, 80% acetonitrile and 0.1% formic acid) at a flow-rate of 0.3 μl/min using the following gradient: 6% B for 5 min; 6%–60% B for 125 min; 60%−100% B for 5 min; hold at 100% B for 5 min; 100%–6% B in 2 min; hold at 6% B for 38 min.

The LTQ-Orbitrap tandem mass spectrometer was operated in a data-dependent mode. Briefly, each full MS scan (60,000 resolving power) was followed by six MS/MS scans where the three most abundant molecular ions were dynamically selected and fragmented by collision-induced dissociation (CID) using a normalized collision energy of 35%; the same three molecular ions were also scanned three times by HCD-MS^2^ with collision energy of 45%. MS scans were acquired in profile mode and MS/MS scans in centroid mode. LTQ-Orbitrap settings were as follows: spray voltage 2.0 kV, 1 microscan for MS1 scans at 60,000 resolution (fwhm at *m*/*z* 400), microscans for MS^2^ at 7500 resolution (fwhm at *m*/*z* 400); full MS mass range, *m*/*z* 400−1400; MS/MS mass range, *m*/*z* 100−2000. The “FT master scan preview mode”, “Charge state screening”, “Monoisotopic precursor selection”, and “Charge state rejection” were enabled so that only the 2+, 3+ and 4+ ions were selected and fragmented by CID and HCD.

### Gene Ontology and KEGG Analysis of Proteomics Data

Functional enrichment analysis were performed using the Database for Annotation, Visualization and Integrated Discovery (DAVID) tool (32) which is a resource consisting of an integrated biological knowledgebase and analytic tools aimed at systematically extracting biological meaning from large gene/protein lists. Using this tool, the proteomic profile of the LTLT-Ca mammospheres versus the LTLT-Ca adherent cells were analyzed and used to identify the Kyoto Encyclopedia of Genes and Genomes (KEGG) pathways and Gene Ontology (GO) terms over-represented by the significantly expressed proteins (genes).

### Molecular Pathway and Network Analysis in Ingenuity Pathway Analysis Software

To systematically evaluate the differences between the LTLT-Ca 3D and LTLT-Ca 2D cells, the proteomic signatures of the two groups were subjected to pathway analysis using IPA software (Ingenuity Systems). Accession numbers and fold changes of proteins from Student**’**s *t*-tests were imported into IPA to identify the biological relationships among the proteins. Canonical pathways and interaction networks were generated based on the knowledge sorted in the Ingenuity Pathway Knowledge base.

### Kaplan Meier (KM) Survival Analysis

The application of KM plot has been described in detail previously (33, 34). Briefly, KM plots were obtained using the KM Plotter web-based (kmplot.com/analysis) curator, which surveys public microarray repositories for relapse free and overall survival among patients with breast, lung, ovarian or gastric cancers. The KM Plotter recognizes 54,675 individual Affymetrix probe sets, and surveys expression data from 4,142 breast cancer patients (as of 2014). Survival and gene expression data were derived from the GEO (Gene Expression Omnibus), TCGA (The Cancer Genome Atlas), and EGA (European Genome-phenome Atlas) databases. Patients were stratified as ER+ or ER-. In order to ascertain midasin expression, Affymetrix probe 212693 was selected. Populations were split by median ERα expression and plots generated accordingly. Relapse Free Survival (RFS) in the total population (2557 patients) was determined.

### Statistical Analysis

Results are expressed as the mean unit ± standard error of the mean (SEM) (***p < 0.001, **p < 0.01, *p < 0.05) using the Graph Pad Prism V.6 software program.

## Results

### Quantitative Proteomic Analysis Reveals Extensive Changes in Protein Expression in Letrozole-Resistant Mammospheres (3D) Compared to Letrozole-Resistant Adherent Cells (2D)

**Figure 1:**
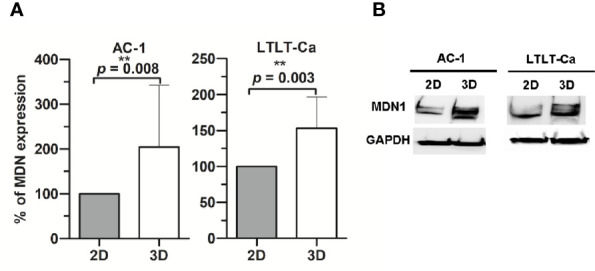
Western Blot analysis of midasin expression. Midasin expression in letrozole-sensitive (AC-1) and letrozole-resistant (LTLT-Ca) breast cancer cells cultured adherently (2D) or as mammospheres (3D). All cells were evaluated by immunoblot to examine the expression of midasin and GAPDH (loading control). **(A)** Graphs depict normalized percentages of protein expression intensities relative to 2D cell counterparts. **(B)** Representative immunoblot depicts the protein expression of midasin and GAPDH.

In order to characterize the proteome of LTLT-Ca cells cultured as CSC-enriched mammospheres (3D) and compare it to their LTLT-Ca adherent cell (2D) counterparts, we performed a gel-free proteomic analysis. This approach combined TMT labeling, two-dimensional HPLC, and high-resolution MS. Over 1,000 proteins with quantitative abundance ratios were identified. Among the quantified proteins, 359 were significantly altered (*p* < 0.05), where 173 were up regulated (**Table 1**) and 186 down regulated (**Table 2**) (*p* < 0.05, fold change >1.20). The most significantly changed protein was a 35-fold increase in midasin, a nuclear chaperone protein required for maturation and nuclear export of the pre-60S ribosomal subunit (15, 17, 35). To validate this finding, western blotting analyses were conducted, and results confirmed that the LTLT-Ca mammospheres (3D) expressed significantly higher levels of midasin compared to the LTLT-Ca adherent cells (2D) (**Figures 1A, B**). A similar result was also observed when letrozole-sensitive AC-1 mammospheres (3D) were compared to letrozole-sensitive AC-1 adherent cells 2D (**Figure 1**). To examine whether increased midasin expression was exclusive to the LTLT-Ca mammospheres, we investigated midasin expression across various cell lines including letrozole-sensitive and letrozole-resistant cell lines. When the 3D mammospheres were compared to their 2D culture counterparts, midasin expression was significantly increased in the letrozole resistant T47D breast cancer cells (T47DaromLR) (**Figure S1A, B**) as well as the MDA-MB-231 triple negative breast cancer cells (**Figure S1C, D**). The MDA-MB-231 mammospheres exhibited an increase in midasin expression greater than 1000% compared to their 2D counterparts which was the most dramatic change in midasin expression. Interestingly, the T47Darom mammospheres expressed significantly lower midasin expression compared to the T47Darom 2D cultures.

**Table 1:** Proteomic analyses of selected up-regulated proteins in LTLT-Ca letrozole-resistant mammospheres where ratios of the fold change (3D/2D) and *p* values are shown.

Accession	Description	# AAs	MW [kDa]	Fold change of LTLT-Ca (3D/2D)	*t*-test (*p* values)
IPI00167941.1	Midasin	5596	632.4	35.049	5.60E-05
IPI00022463.2	Serotransferrin	698	77.0	10.988	3.22E-05
IPI00871227.1	Isoform 1 of Hemicentin-1	5635	613.0	8.160	3.95E-04
IPI00942032.1	Anterior gradient protein 2 homolog	175	20.0	7.932	6.77E-08
IPI00073772.6	Fructose-1,6-bisphosphatase 1	338	36.8	3.974	8.18E-05
IPI00976367.1	Uncharacterized protein	76	9.0	3.408	3.07E-05
IPI00028376.1	Mitochondrial import inner membrane translocase subunit Tim8 A	97	11.0	2.971	3.14E-04
IPI00465352.2	Calcyphosin	189	21.0	2.626	2.47E-04
IPI00017526.1	Protein S100-P	95	10.4	2.717	1.04E-03
IPI00301058.5	Vasodilator-stimulated phosphoprotein	380	39.8	2.382	5.95E-05
IPI00646689.1	Thioredoxin domain-containing protein 17	123	13.9	2.482	5.28E-05
IPI00292020.3	Spermidine synthase	302	33.8	2.306	3.35E-06
IPI00785061.1	Isoform 2 of Membrane metallo-endopeptidase-like 1	611	69.9	2.173	3.89E-04
IPI00106687.4	Latexin	222	25.7	2.188	8.40E-05
IPI00304409.3	Calcium-regulated heat stable protein 1	147	15.9	2.344	2.06E-03
IPI00010182.4	Isoform 1 of Acyl-CoA-binding protein	87	10.0	2.335	3.99E-03
IPI00745466.1	Isoform ARPP-16 of cAMP-regulated phosphoprotein 19	96	10.6	2.141	4.50E-03
IPI00012197.1	dCTP pyrophosphatase 1	170	18.7	1.870	2.45E-03
IPI00964927.1	UDP-glucose 6-dehydrogenase isoform 3	397	44.4	1.921	1.58E-04
IPI00003935.6	Histone H2B type 2-E	126	13.9	1.865	1.26E-04
IPI00418262.5	Fructose-bisphosphate aldolase	451	48.4	2.181	7.56E-04
IPI00916111.3	Malate dehydrogenase, cytoplasmic	334	36.4	1.862	4.57E-05
IPI00021828.1	Cystatin-B	98	11.1	1.978	1.69E-03
IPI00883944.1	Gamma-synuclein	79	8.3	1.690	1.98E-03
IPI00953696.1	glutathione reductase, mitochondrial isoform 4 precursor	440	47.2	1.809	8.69E-05
IPI00301434.4	BolA-like protein 2	152	16.9	1.604	9.27E-03
IPI00843765.1	Isoform 3 of Spectrin alpha chain, brain	2452	282.1	1.798	1.14E-03
IPI00853358.1	MUC1 isoform T9	110	12.1	1.616	7.89E-04
IPI00062037.1	Dynein light chain 2, cytoplasmic	89	10.3	1.745	1.42E-06
IPI00218829.9	Eukaryotic peptide chain release factor GTP-binding subunit ERF3A	499	55.7	1.647	1.27E-04
IPI00827491.1	Isoform 2 of Ubiquitin-like modifier-activating enzyme 6	578	65.7	1.711	5.60E-05
IPI01012504.1	6-phosphogluconate dehydrogenase, decarboxylating	470	51.8	1.543	3.61E-03
IPI00426077.4	Isoform 2 of Calcineurin-like phosphoesterase domain-containing protein 1	172	19.0	1.737	4.85E-05
IPI00465439.5	Fructose-bisphosphate aldolase A	364	39.4	1.683	1.55E-04
IPI00414315.10	Isoform 1 of Epidermal growth factor receptor kinase substrate 8-like protein 2	715	80.6	1.561	5.92E-04
IPI00027933.3	Proteasome subunit beta type-10	273	28.9	1.620	2.83E-03
IPI00219446.5	Phosphatidylethanolamine-binding protein 1	187	21.0	1.677	1.54E-04
IPI00419585.9	Peptidyl-prolyl cis-trans isomerase A	165	18.0	1.585	2.12E-06
IPI00007052.6	Mitochondrial fission 1 protein	152	16.9	1.536	4.54E-05
IPI00979393.2	Isoform 3 of Calpastatin	590	63.6	1.607	4.78E-03
IPI00027223.2	Isocitrate dehydrogenase [NADP] cytoplasmic	414	46.6	1.515	6.23E-05
IPI00396485.3	Elongation factor 1-alpha 1	462	50.1	1.460	1.93E-03
IPI00411704.9	Isoform 1 of Eukaryotic translation initiation factor 5A-1	154	16.8	1.565	1.79E-03
IPI00219953.5	UMP-CMP kinase isoform a	228	25.8	1.512	1.68E-03
IPI00794609.1	Cysteine synthase	177	19.1	1.457	1.32E-04
IPI00015018.1	Inorganic pyrophosphatase	289	32.6	1.514	1.24E-04
IPI00398922.4	Protein phosphatase 1 regulatory subunit 14B	199	21.0	1.564	9.47E-03
IPI00220637.5	Seryl-tRNA synthetase, cytoplasmic	514	58.7	1.412	4.43E-03
IPI00221362.3	Isoform 3 of Apoptosis-associated speck-like protein containing a CARD	135	15.0	1.337	7.02E-03
IPI00815656.1	Parathymosin (Fragment)	63	7.4	1.700	2.33E-02
IPI00903145.1	Radixin	583	68.5	1.410	3.09E-04
IPI00295386.7	Carbonyl reductase [NADPH] 1	277	30.4	1.367	5.76E-03
IPI00025285.3	V-type proton ATPase subunit G 1	118	13.7	1.250	1.03E-01
IPI00028091.3	Actin-related protein 3	418	47.3	1.397	1.51E-03
IPI00479186.7	Isoform M2 of Pyruvate kinase isozymes M1/M2	531	57.9	1.428	5.13E-06
IPI00028004.2	Proteasome subunit beta type-3	205	22.9	1.289	1.32E-02
IPI00797270.4	Isoform 1 of Triosephosphate isomerase	249	26.7	1.402	1.10E-05
IPI00375513.3	Isoform Soluble of Catechol O-methyltransferase	221	24.4	1.513	1.99E-03
IPI00294398.2	Isoform 1 of Hydroxyacyl-coenzyme A dehydrogenase, mitochondrial	314	34.3	1.270	9.69E-03
IPI00465436.4	Catalase	527	59.7	1.353	1.84E-04
IPI00219018.7	Glyceraldehyde-3-phosphate dehydrogenase	335	36.0	1.357	1.54E-04
IPI00216691.5	Profilin-1	140	15.0	1.319	8.52E-04
IPI00298547.3	Protein DJ-1	189	19.9	1.410	4.13E-03
IPI01011912.1	Phosphoglycerate kinase	389	41.4	1.330	8.14E-05
IPI00026781.3	Fatty acid synthase	2511	273.3	1.340	1.44E-03
IPI00217966.9	Isoform 1 of L-lactate dehydrogenase A chain	332	36.7	1.276	4.72E-04
IPI01011075.1	Superoxide dismutase	162	18.3	1.386	3.51E-03
IPI00479145.3	Keratin, type I cytoskeletal 19	400	44.1	1.364	3.25E-04
IPI00016610.2	Poly(rC)-binding protein 1	356	37.5	1.337	1.58E-02
IPI00011229.1	Cathepsin D	412	44.5	1.254	1.33E-03
IPI00218070.1	Isoform GRB3-3 of Growth factor receptor-bound protein 2	176	20.5	1.505	6.20E-03
IPI00645307.2	Isoform 1 of Isopentenyl-diphosphate Delta-isomerase 1	227	26.3	1.303	1.55E-03
IPI00218482.2	Isoform Short of ES1 protein homolog, mitochondrial	237	24.7	1.297	1.10E-03
IPI00011416.2	Delta(3,5)-Delta(2,4)-dienoyl-CoA isomerase, mitochondrial	328	35.8	1.389	7.22E-03
IPI00014808.1	Platelet-activating factor acetylhydrolase IB subunit gamma	231	25.7	1.240	5.28E-03
IPI00028109.1	Protein dpy-30 homolog	99	11.2	1.257	4.62E-03
IPI00908881.3	Glucose-6-phosphate isomerase	531	60.0	1.241	8.26E-03
IPI00010896.3	Chloride intracellular channel protein 1	241	26.9	1.257	1.66E-04
IPI00147874.1	Sialic acid synthase	359	40.3	1.330	5.10E-04
IPI00218493.7	Hypoxanthine-guanine phosphoribosyltransferase	218	24.6	1.193	6.98E-02
IPI00981623.1	Uncharacterized protein	98	10.6	1.215	1.97E-02
IPI00377066.1	ras suppressor protein 1 isoform 2	224	25.5	1.209	5.05E-02
IPI00472003.1	Isoform 4 of BH3-interacting domain death agonist	99	11.3	1.268	3.76E-04
IPI00216770.1	Isoform 2 of 26S protease regulatory subunit 6B	387	43.5	1.131	2.17E-01
IPI00550069.3	Ribonuclease inhibitor	461	49.9	1.261	8.96E-07

**Table 2:** Proteomic analyses of selected down-regulated proteins in LTLT-Ca letrozole-resistant mammospheres where ratios (3D/2D) of the fold changes and *p* values are shown.

Accession	Description	# AAs	MW [kDa]	Fold change (3D/2D)	*t*-test (*p* values)
IPI00013485.3	40S ribosomal protein S2	293	31.3	-1.704	7.75E-05
IPI00012750.3	40S ribosomal protein S25	125	13.7	-1.780	4.94E-06
IPI00221091.9	40S ribosomal protein S15a	130	14.8	-1.784	2.22E-04
IPI00216514.6	Isoform OA3-293 of Leukocyte surface antigen CD47	292	31.7	-1.784	4.89E-05
IPI00647168.1	Ribosomal protein L11	131	14.9	-1.703	1.29E-04
IPI00215780.5	40S ribosomal protein S19	145	16.1	-1.782	2.92E-04
IPI00026271.5	40S ribosomal protein S14	151	16.3	-1.746	6.69E-05
IPI00908521.1	Isoform 2 of Brain acid soluble protein 1	173	17.7	-1.673	2.78E-03
IPI00644936.1	Isoform 3 of Guanine nucleotide-binding protein G(s) subunit alpha isoforms short	379	44.2	-2.055	2.63E-05
IPI00926712.1	Integrin beta	681	74.9	-2.021	3.96E-08
IPI00011253.3	40S ribosomal protein S3	243	26.7	-1.849	1.43E-04
IPI00019472.4	Neutral amino acid transporter B(0)	541	56.6	-2.039	8.05E-06
IPI00979226.1	60S ribosomal protein L17 isoform b	146	17.1	-2.004	8.59E-05
IPI00337335.8	Isoform 1 of Myosin-14	1995	227.7	-1.967	7.32E-05
IPI00922108.2	integrin alpha-V isoform 2	1002	111.1	-2.111	8.17E-06
IPI00645589.1	Isoform 3 of Choline transporter-like protein 2	704	79.8	-2.089	5.47E-05
IPI00985384.1	ATP-dependent RNA helicase DDX3X isoform 3	646	71.3	-2.194	6.26E-05
IPI00008438.1	40S ribosomal protein S10	165	18.9	-2.337	5.48E-07
IPI00396321.1	Leucine-rich repeat-containing protein 59	307	34.9	-2.144	5.22E-05
IPI00015842.1	Reticulocalbin-1	331	38.9	-2.136	2.76E-04
IPI00221088.5	40S ribosomal protein S9	194	22.6	-2.467	7.90E-04
IPI00455315.4	Isoform 1 of Annexin A2	339	38.6	-2.496	5.64E-05
IPI00009865.4	Keratin, type I cytoskeletal 10	584	58.8	-2.176	7.64E-02
IPI00297910.2	Tumor-associated calcium signal transducer 2	323	35.7	-3.207	9.52E-06

Additionally, we observed increased expression of S100-P, Profilin-1, Elongation factor 1α1, and serotransferrin. In our previous report, letrozole resistant cells (LTLT-Ca) were compared to letrozole-sensitive cells (AC-1) and a global proteomic signature was identified where several proteins were increased in the letrozole-resistant LTLT-Ca adherent 2D cells (ie. Protein S100-P and Pofilin-1) versus AC-1 adherent 2D cells (6). Here, those proteins are further increased in the LTLT-Ca 3D mammospheres versus 2D adherent cells.

### Letrozole Resistant Mammospheres Are Associated With Increased Components of the Translational Machinery

**Figure 2:**
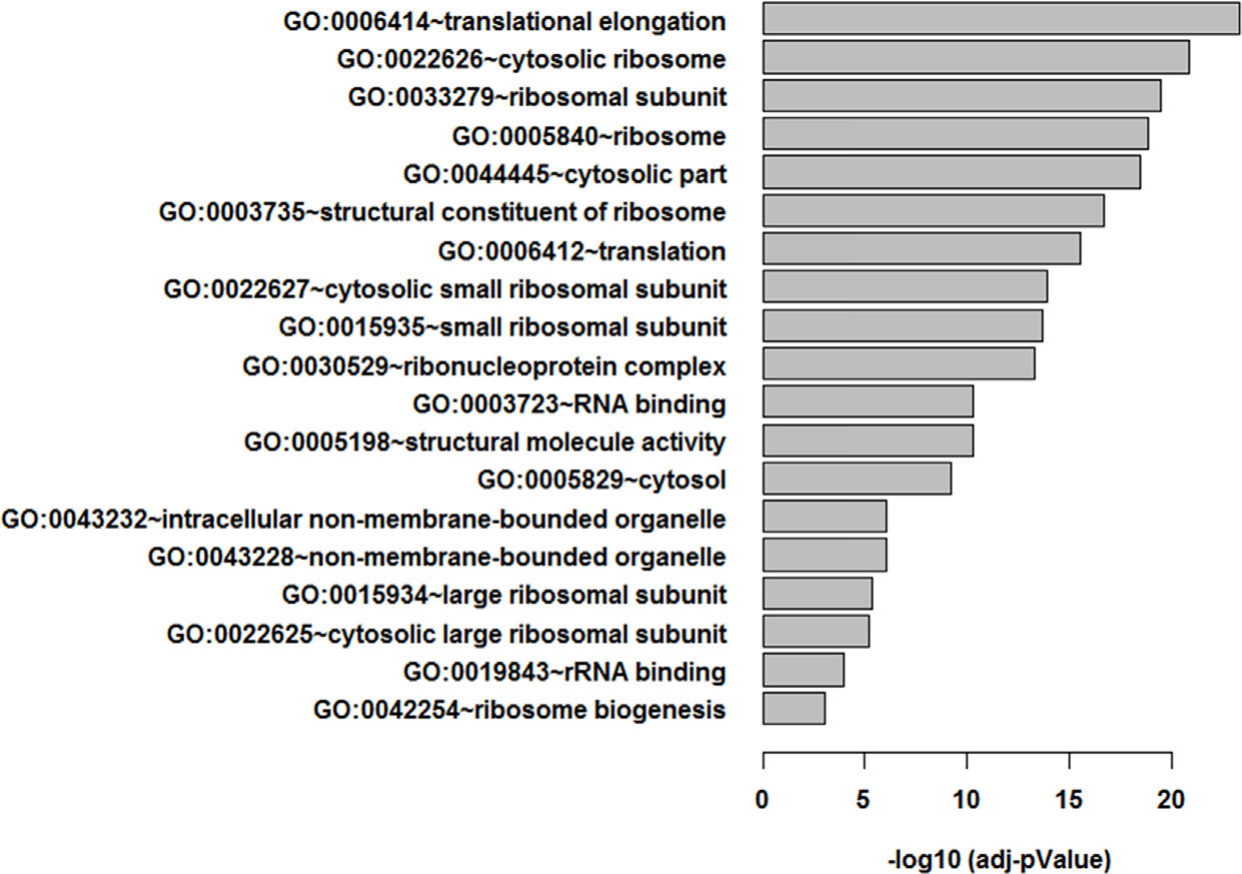
Functional enrichment analysis of LTLT-Ca proteome. Functional enrichment analysis were performed using the DAVID tool (32), resulting in 19 gene ontology (GO) terms.

**Figure 3:**
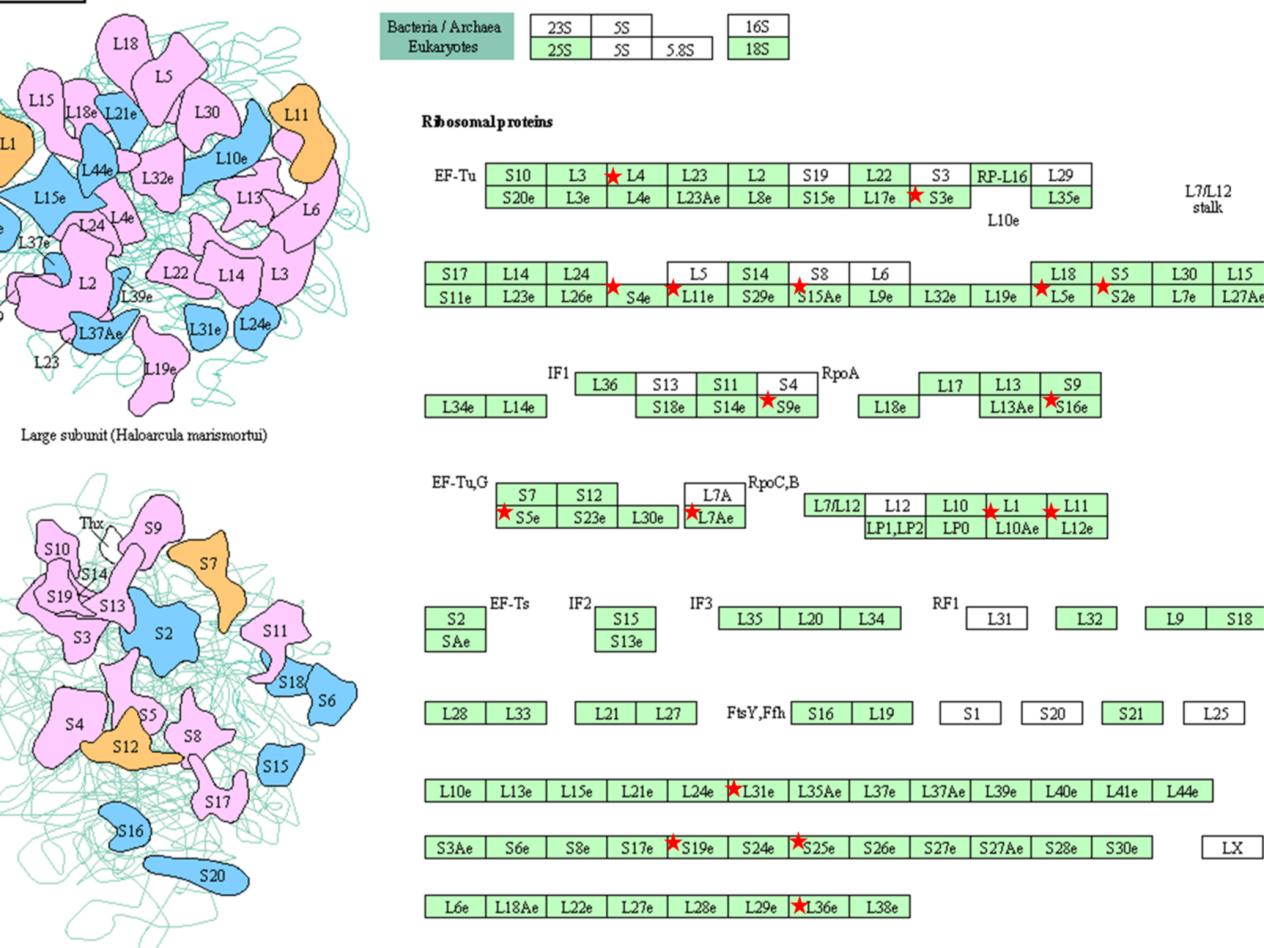
KEGG pathway (hsa03010:Ribosome) Graphs. KEGG pathway were over-represented (BH adjusted *p* value < 0.01) by the top 65 down-regulated (BH adjusted p-value < 0.01) proteins. The pathway hsa03010:Ribosome was graphically represented and the ribosomal proteins were marked with red stars. The graphics were retrieved from the output of the functional enrichment analysis using the DAVID tool. The bar plot was generated by our lab-owned R codes.

An additional analysis of the proteomes was performed using the Benjamini-Hochberg (BH) method. The selection was based on the following criteria: (1) the adjusted *p* value < 0.01 (corrected using the BH method) (36) and (2) the average expression ratio greater than 1.5 for upregulated proteins or less than 0.67 for down-regulated proteins. Using the more stringent BH method, we identified 55 upregulated proteins (**Table S1**) and 65 downregulated proteins (**Table S2**) in the 3D LTLT-Ca mammospheres compared to the 2D adherent cells. Using this data, functional enrichment analyses were conducted utilizing the DAVID tool (http://david.abcc.ncifcrf.gov/) (32). Based on the result, 19 GO terms (**Figure 2** and **Table S3**) and one KEGG pathway (hsa03010:Ribosome) were over-represented (BH adjusted p value < 0.01) by the top 65 downregulated (BH adjusted p-value < 0.01) proteins. The KEGG term was enriched 29-fold (**Table S4**). Several of these GO terms including “translational elongation”, “ribosomal subunit”, “cytosolic ribosome” and “translation” was enriched by over 15-fold and of the 19 GO terms identified, 15 were related to protein synthesis and ribosomes. Interestingly, the upregulated protein set was less informative. Only one cellular component GO term “cytosol” and one biological process GO term “response to organic cyclic substance” were over-represented by the proteins. Our findings of increased midasin expression coupled with the GO and KEGG analyses, supported the previously identified role of midasin. Further, many of the significantly altered proteins shown in **Table 2** were ribosomal proteins associated with the 40S and 60S ribosomes (highlighted with red stars in **Figure 3**). While several of the 40S and 60S ribosomal proteins were significantly altered, more of the 40S ribosomal proteins were decreased compared to the 60S ribosomal proteins (**Table S2**). The downregulated proteins associated with the 40S ribosomal subunit included, RPS9, RPS10, and RPS14, among others, while those associated with the 60S subunit included RPL11, RPL10a, and RPL7a. Taken together, these findings suggest increased midasin expression in CSC-enriched letrozole-resistant mammospheres is highly associated with alterations in the translational machinery impacting the composition of ribosomal proteins, which may contribute to endocrine resistance.

**Figure 4:**
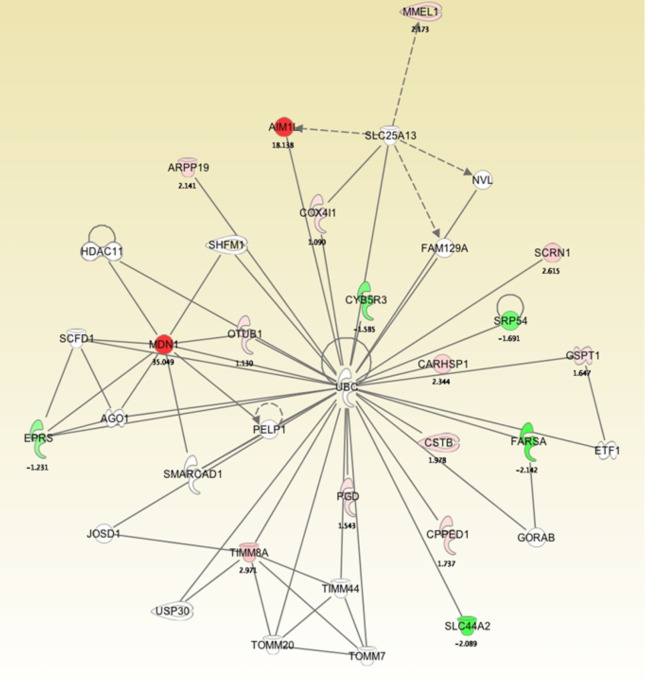
Molecular pathway and network analysis of proteomics analysis of differentially expressed proteins in LTLT-Ca mammospheres and LTLT-Ca adherent cells. The proteome data was uploaded into IPA software that determined a network view of predicted associations for midasin. The network nodes represent proteins, and solid lines indicated a direct relationship while dashed lines represent an indirect relationship between proteins.

To develop a network view of predicted protein associations, a molecular pathway and network analysis using IPA software was used with the differentially regulated proteins identified by MS (**Figure 4**). The resulting network demonstrated that midasin has a direct interaction with the ubiquitin thioesterase (OTUB1) with a 1.13-fold increase. This finding was interesting as OTUB1 inhibits the degradation of FOXM1 transcription factor, typically upregulated and overexpressed in aggressive therapy resistant breast cancer (37). Additionally, midasin directly interacted with glutamyl-prolyl-tRNA synthetase (EPRS), with a -1.23-fold decrease. EPRS is responsible for charging tRNAs with their cognate amino acids and is reported in the regulation of breast tumorigenesis (38). On the other hand, midasin indirectly interacted with calcium-regulated heat stable protein 1 (CARHSP1) and phenylalanyl-tRNA synthetase subunit alpha (FARSA) through direct interactions with ubiquitin C (UBC). CARHSP1 binds and regulates the stability of target mRNAs and increased by 2.34-fold, while FARSA is involved in charging tRNAs with their cognant amino acids and decreased by 2.14-fold. These direct and indirect protein interactions with midasin shed additional light on the inherent complexity of the proteome of CSC-enriched letrozole-resistant breast cancer.

### Increased Midasin Expression in Breast Cancer Tissue

**Figure 5:**
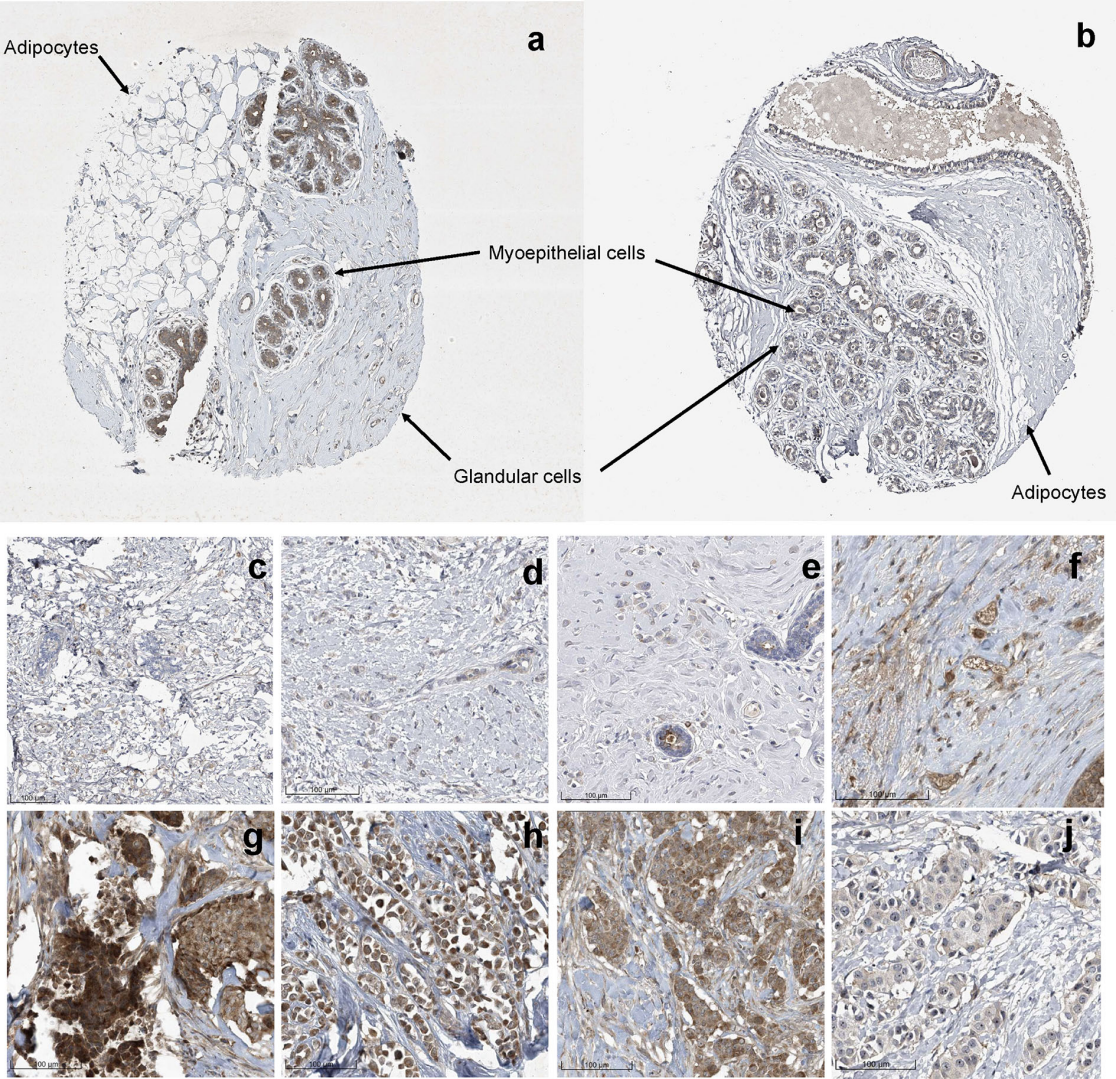
Increased Midasin Expression Profile in Normal Breast and Breast Cancer Samples. Representative protein staining of midasin protein expression in normal mammary tissue **(A, B)**, lobular carcinoma tissue **(C–F)**, and ductal carcinoma tissue **(G–J)**. Image credit: Human Protein Atlas, www.proteinatlas.org, (Uhlén et al, 2015). Image available at the following URL: v19.proteinatlas.org/humancell.

Previously, Uhlén et al., mapped the human tissue proteome using quantitative transcriptomics at the tissue and organ level, combined with tissue microarray-based immunohistochemistry (39). The data was integrated into an interactive web-based database allowing exploration of individual proteins and global expression patterns in all major tissues and organs of the human body. The database serves as a repository for immunohistochemistry tissue samples from breast cancer patients. This database was mined to further explore the expression pattern of midasin in normal breast tissue (**Table 3**) and breast cancer tissue samples (**Table 4**). In normal breast tissue, the adipocytes, glandular, and myoepithelial cells were examined for midasin expression (**Figures 5A, B**) and in most instances the tissues stained weakly for midasin. Likewise, when lobular carcinoma tissue samples were examined, the majority of the samples exhibited weak staining intensity while one sample expressed moderate staining intensity (**Figures 5C–F**). However, the majority of ductal carcinoma tissue samples expressed moderate staining intensity (**Figures 5G–J**). Regardless of the tissue type examined (ie., normal or cancerous), midasin was predominately localized to the cytoplasm and membrane. As such, the immunohistochemistry analyses confirmed that midasin is ubiquitously expressed in both normal breast tissue and cancer tissue, but ductal carcinoma tumor tissue had a higher midasin staining intensity.

**Table 3:** Normal breast tissue sample profile.

Patient Sample Identification	Sample type	Intensity	Subcellular localization	Fraction of cells stained	Age
Patient 2773	Adipocytes	weak	Cytoplasmic/membranous	<25%	23
	Glandular	moderate	Cytoplasmic/membranous, nuclear	>75%	23
	Myoepithelial	weak	Cytoplasmic/membranous	>75%	23
Patient 3544	Adipocytes	negative	Cytoplasmic/membranous	none	45
	Glandular	weak	Cytoplasmic/membranous Nuclear	25–75%	45
	Myoepithelial	weak	Cytoplasmic/membranous	25–75%	45

Normal breast tissue profile depicting sample type, staining intensity, subcellular localization, fraction of cells stained, and patient age.

**Table 4:** Breast cancer patient tumor tissue profile.

Patient Sample Identification	Sample type	Staining Intensity	Subcellular localization	Fraction of cells stained	Age
Patient 2252	Lobular carcinoma	weak	Cytoplasmic/membranous	<25%	47
Patient 2898	Lobular carcinoma	weak	Cytoplasmic/membranous, nuclear	25–75%	41
Patient 3535	Lobular carcinoma	weak	Cytoplasmic/membranous	25–75%	47
Patient 2565	Lobular carcinoma	moderate	Cytoplasmic/membranous	>75%	51
Patient 2428	Ductal carcinoma	moderate	Cytoplasmic/membranous	>75%	75
Patient 1910	Ductal carcinoma	moderate	Cytoplasmic/membranous	>75%	61
Patient 2091	Ductal carcinoma	moderate	Cytoplasmic/membranous	>75%	40
Patient 3257	Ductal carcinoma	weak	Nuclear	25–75%	39

Tumor sample profile depicting sample type, staining intensity, subcellular localization, fraction of cells stained, and patient age.

### High Midasin Expression Is Positively Correlated With Decreased RFS in ER- Breast Cancer Patients

**Figure 6:**
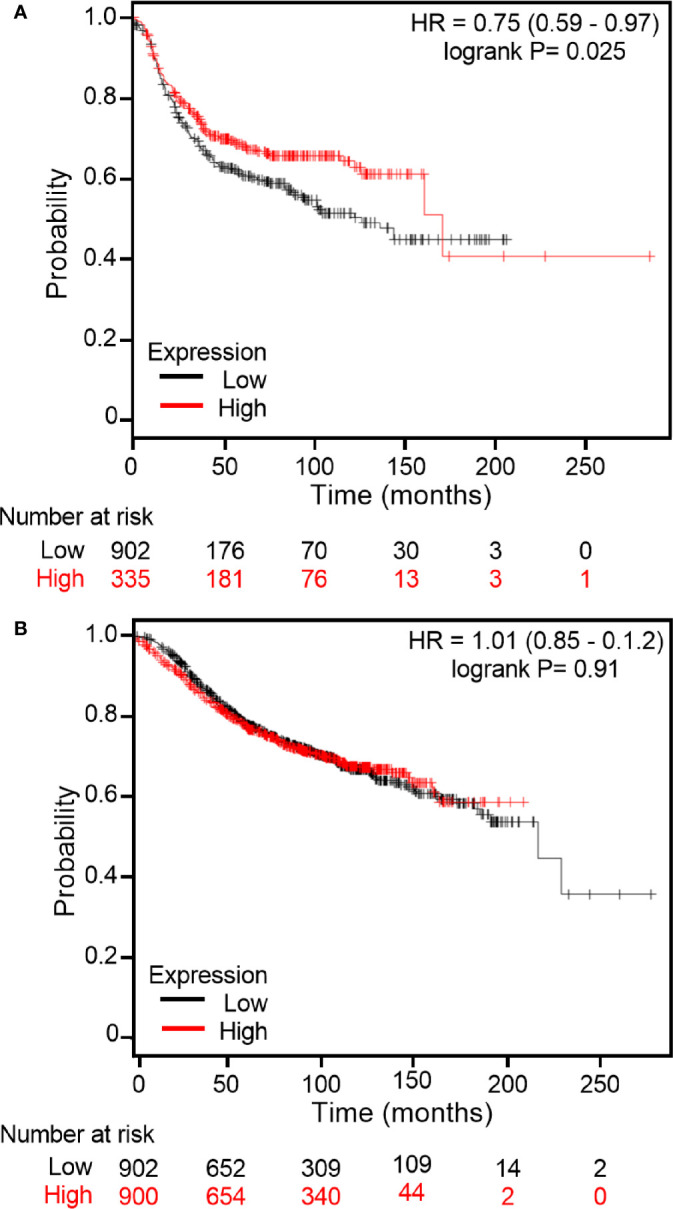
Kaplan Meier plots of RFS based on midasin expression in ER+ and ER- breast cancer patients. Using Kaplan Meier Plotter, publicly available microarray repositories for breast cancer were interrogated to determine whether midasin expression was associated with different survival rates among **(A)** Estrogen Receptor negative (ER-) and **(B)** Estrogen Receptor positive (ER+) breast cancer patients. Hazard ratio (HR) and Logrank P values are shown. Low midasin expression (below median) is noted in black, and the high midasin expression (above median) is noted in red.

As immunohistochemistry results demonstrated that some breast cancer tissue expressed higher levels of midasin compared to normal breast tissue samples, it was important to examine the clinical relevance of midasin levels on patient survival and determine within this context whether hormone receptor status was relevant to patient outcomes. To predict whether midasin expression was associated with differences in RFS, KM Plotter was used to interrogate publicly available microarray repositories for ER+ and ER- breast cancer patients. We chose to stratify patients based on hormone receptor status because our LTLT-Ca model of letrozole resistance is hormone independent (40) and reflects patients within this population as they progress to metastatic disease. Based on these parameters, high midasin expression was associated with a significant decrease in RFS among ER- patients (p = 0.0214) (**Figure 6A**) whereas increased midasin expression did not result in a statistically significant difference in RFS among ER+ patients (**Figure 6B**). Altogether, we demonstrate for the first time, a global proteomic signature for cancer stem cell enriched letrozole resistant mammospheres that is associated with high midasin expression, increased components of the translational machinery, and finally, that increased midasin expression is associated with decreased RFS in ER- breast cancer patients.

## Discussion

Overcoming aromatase inhibitor resistance continues to be a challenge clinically, further compounded by the radiation and chemotherapy resistant nature of breast CSCs. It has been shown that culturing cells as mammospheres allows for the propagation of breast cancer stem cells (41). This is critical as 3D cultures are more physiologically relevant than 2D cultures and mammospheres and adherent cells possessed markedly different proteomes (42). To this end, whole MCF-7 mammospheres, as well as subpopulations within spheres, have been shown to be more tumorigenic than 2D monolayer parental cultures (43–45). This suggests an enriched population of breast CSCs within spheres may be partly responsible for conferring tumorigenesis. To gain a deeper understanding of the mechanism that drives AI-resistant breast cancer, previous studies from our lab compared the proteome of letrozole-sensitive AC-1 breast cancer cells to letrozole-resistant LTLT-Ca breast cancer cells, where both cell lines were cultured adherently (2D). We identified a global proteomic signature associated with hormone independence, enhanced cell motility, and EMT among the 1,743 differentially regulated proteins (6). However, one of the limitations of this previous study was evaluating cells cultured as monolayers (2D) may have prevented the identification of proteins expressed when cells are cultured 3D as CSC-enriched mammospheres. Therefore, in this current study, we evaluated the proteome of the letrozole-resistant CSC-enriched mammosphere population and compared it to letrozole-resistant adherent cells to identify additional candidate proteins involved in endocrine resistance.

Here, we identified a novel proteomic signature associated with translation that stemmed from the observation that midasin was upregulated by 35-fold in LTLT-Ca mammospheres (**Table 1**, **Figure 1**). This was a unique feature of this signature as midasin was not detected when the 2D LTLT-Ca cells were compared to 2D AC-1 cells (6). The present finding of increased midasin expression in cancer stem cell enriched mammospheres is highly relevant, as midasin plays a key role in driving nuclear export of pre-ribosomal particles through removal of biogenesis factors at critical checkpoints of 60S ribosome assembly (46, 47). In addition, previous studies found that midasin functions as a nuclear chaperone, is involved in the assembly/disassembly of macromolecular complexes in the nucleus, and is associated with maturation of 60S ribosome subunits (15). Our immunohistochemistry analysis showed that midasin was ubiquitously expressed in normal breast tissue as well as breast tumor tissue (**Figure 5**), suggesting its importance in normal physiologic functions. Interestingly, midasin levels were increased in ductal carcinoma compared to lobular carcinoma, but without additional tumor features such as molecular classification (hormone receptor status), tumor grade, node status, and tumor stage the exact relevance of this finding is yet to be understood. While the precise role of midasin in cancer has yet to be determined, the robust increase in protein expression observed in the mammospheres, the expression of midasin in ductal carcinoma tissue samples, along with its involvement in RFS, supports its involvement in both endocrine sensitive and endocrine resistant breast cancer.

Since ribosomal proteins play significant roles in normal cell functions, small and large ribosomal subunit deficiencies lead to distinct gene expression signatures that alter cell growth rates (48). This deregulation or altered expression is harmful to the integrity and maintenance of the cell, causing serious consequences for cell fate. Some of the reported consequences on this ribosomal protein deregulation include defects in the synthesis of proteins relevant for tumorigenesis or cell survival, changes that affect the translation of specific mRNAs (ie., oncogenes and/or tumor suppressor genes), and alterations in the availability of ribosomes. To further investigate the protein expression profile of CSC-enriched letrozole resistant breast cancer cells, we conducted bioinformatics analyses. Our results show that many of the downregulated proteins were small ribosomal proteins associated with GO terms affiliated with the translational machinery (**Table 2**, **Figure 2**). Interestingly, when the down-regulated proteins were adjusted to control for false discovery rates (**Table S2**), the 40S small ribosomal proteins exhibited the greatest reduction in fold change compared to the 60S large ribosomal proteins. In a previous report, Fang and Zhang used integrated bioinformatics analyses and identified that low mRNA expression of RPL11, RPS14, RPS9, and RPL10A were related to a worse overall survival of breast cancer patients (49). This was notable since, in this study, we also observed decreased expression of RPL11 (-1.7-fold), RPS14 (-1.7-fold), and RPS9 (-2.5-fold) in the letrozole resistant mammospheres (**Figure 3**). While the role of RPS9 in breast cancer is unknown, decreased RPS9 expression has been observed in other solid tumors including pancreatic cancer (50).

It is well known that many cells transitioning from hormone dependent to hormone-refractory states no longer require estrogen for growth as they adapt and utilize MAPK and/or EGFR signaling pathways (6, 51). However, there may be other unknown regulatory mechanisms contributing to hormone independence. Here, we observed that midasin interacts with two downregulated proteins, EPRS and FARSA (**Figure 4**). Decreased expression of both proteins can affect transferring an amino acid from the tRNA onto a growing peptide and alter translation of specific proteins. While EPRS is necessary for the proliferation of tamoxifen-resistant ER+ breast cancer, its role in ER- breast cancer is unclear (37). Likewise, while a specific role for FARSA in cancer has not been identified, dysregulation of aminoacyl tRNA synthetases contribute to initiation, maintenance, and progression of carcinogenesis (52). Since the rate of cell growth has been demonstrated to be proportional to ribosomal biogenesis (53), it is likely that increased midasin and altered ribosomal protein expression could prime the translational machinery for increased protein synthesis or stabilize key proteins involved in cell proliferation.

It is well established that alterations in ribosomal proteins’ expression could result in alterations in cellular homeostasis, affecting protein synthesis that could ultimately lead to cancer initiation or progression. To this end, it was critical to assess the impact of midasin expression levels on RFS in the ER+ and ER- breast cancer patients (**Figure 6**). There was no statistically significant difference in RFS within the ER+ cohort. However, high levels of midasin were more relevant in the ER- cohort and resulted in decreased RFS. For the first two years, the survival curves overlapped in the ER- cohort and initially, high levels of midasin seemed to be protective by extending survival up to an additional 15 years. However, beyond 15 years, high levels of midasin were associated with a worse prognosis among the ER- cohort, suggesting an early protective role for midasin within this population. It should be noted that although there was a trend in the association between high midasin levels and decreased RFS beyond 15 years, there were only 6 patients and a more robust sample size is needed to determine if this trend persists. While the precise role of midasin on survival is still not fully studied, its potential as a biomarker is very promising.

While the role of many of these AAA enzymes in breast cancer remains elusive, recent progress has revealed that midasin functions at successive maturation steps to remove ribosomal factors at critical transition points, first driving the exit of the early pre-60S particles from the nucleolus and then driving late pre-60S particles from the nucleus to the cytoplasm (46, 54). Ribosome biogenesis remains at the heart of translation thus requiring extensive regulation and coordination to meet the cellular demands of continuous ribosome production. As such, disruption of protein expression and interactions involved in ribosomal biogenesis affects translation, hence the overall fitness of the cell. This indicates that future studies are required to unravel a detailed mechanistic understanding of ribosome biogenesis which, in part, could aid in developing new strategies for targeting cancer stem cell enriched AI-resistant breast cancer.

## Data Availability Statement

The proteomic dataset is available at the Peptide Atlas repository through the link: https://db.systemsbiology.net/sbeams/cgi/PeptideAtlas/PASS_View?identifier=PASS01549.

## Author Contributions

KG and SL directed the project, conceived, and designed the experiments with supervision of ST. SL JP, RW, AD, WZ, and KZ contributed to data acquisition. WZ, KZ, and KG analyzed and interpreted the data. ST wrote the manuscript with support from KG. RW, JP, AD, and KG proofread the manuscript. All authors contributed to the article and approved the submitted version.

## Funding

This work was also supported in part by NIH grant SC1GM125617 awarded to ST. This publication was also made possible by funding from the Louisiana Cancer Research Consortium (LCRC) and the NIH-RCMI grant G12MD007595 from the National Institute on Minority Health and Health Disparities (NIMHD). The publication was also funded by NIH-RCMI grant U54MD007582 from the NIMHD. The contents are solely the responsibility of the authors and do not necessarily represent the official views of the LCRC or the NIH. We also like to thank the Xavier University of Louisiana Cell and Molecular Biology Core Facility.

## Conflict of Interest

The authors declare that the research was conducted in the absence of any commercial or financial relationships that could be construed as a potential conflict of interest.

## Supplementary Material

Figure S1 Western Blot analysis of midasin expression. Immunoblots of midasin protein expression in letrozole-sensitive (T47Darom), letrozole-resistant (T47DaromLR) breast cancer cells and MDA-MB 231 triple negative breast cancer cells cultured adherently (2D) or as mammospheres (3D). All cells were evaluated by immunoblot to examine the expression of midasin and GAPDH (loading control). **(A**, **C)** Graphs depict normalized percentages of protein expression intensities relative to 2D cell counterparts. **(B**, **D)** Representative immunoblot depicts the protein expression of midasin and GAPDH.

Table S1 Proteomic analysis using BH method of upregulated proteins in LTLT-Ca mammospheres where the adjusted p value < 0.01.

Table S2 Proteomic analysis using BH method of downregulated proteins in LTLT-Ca mammospheres where the adjusted p value < 0.01. Large ribosomal proteins are indicated by red bold text and small ribosomal proteins are indicated by blue bold text.

Table S3 Gene Ontology analysis of the proteomic data of LTLT-Ca mammospheres.

Table S4 KEGG Analysis of Proteomics data.

Table S5 Letrozole Resistant Proteomic Analysis. Using a gel free proteomic approach combining TMT labeling, two-dimensional HPLC, and high-resolution mass spectrometry, over 1000 proteins were identified with quantitative abundance ratios present in both the LTLT-Ca mammospheres and the LTLT-Ca adherent breast cancer cells. Of the quantified proteins, 359 were significantly altered (p < 0.05), where 173 were upregulated (shown in yellow) and 186 downregulated shown in purple (p < 0.05, fold change >1.20).
