# Supplementary figures and images for: Quantitative Proteomic Profiling Identifies a Potential Novel Chaperone Marker in Resistant Breast Cancer
Source: Front Oncol. 2021 Feb 25;11:540134. doi: 10.3389/fonc.2021.540134 (PMC7951058; doi:10.3389/fonc.2021.540134)

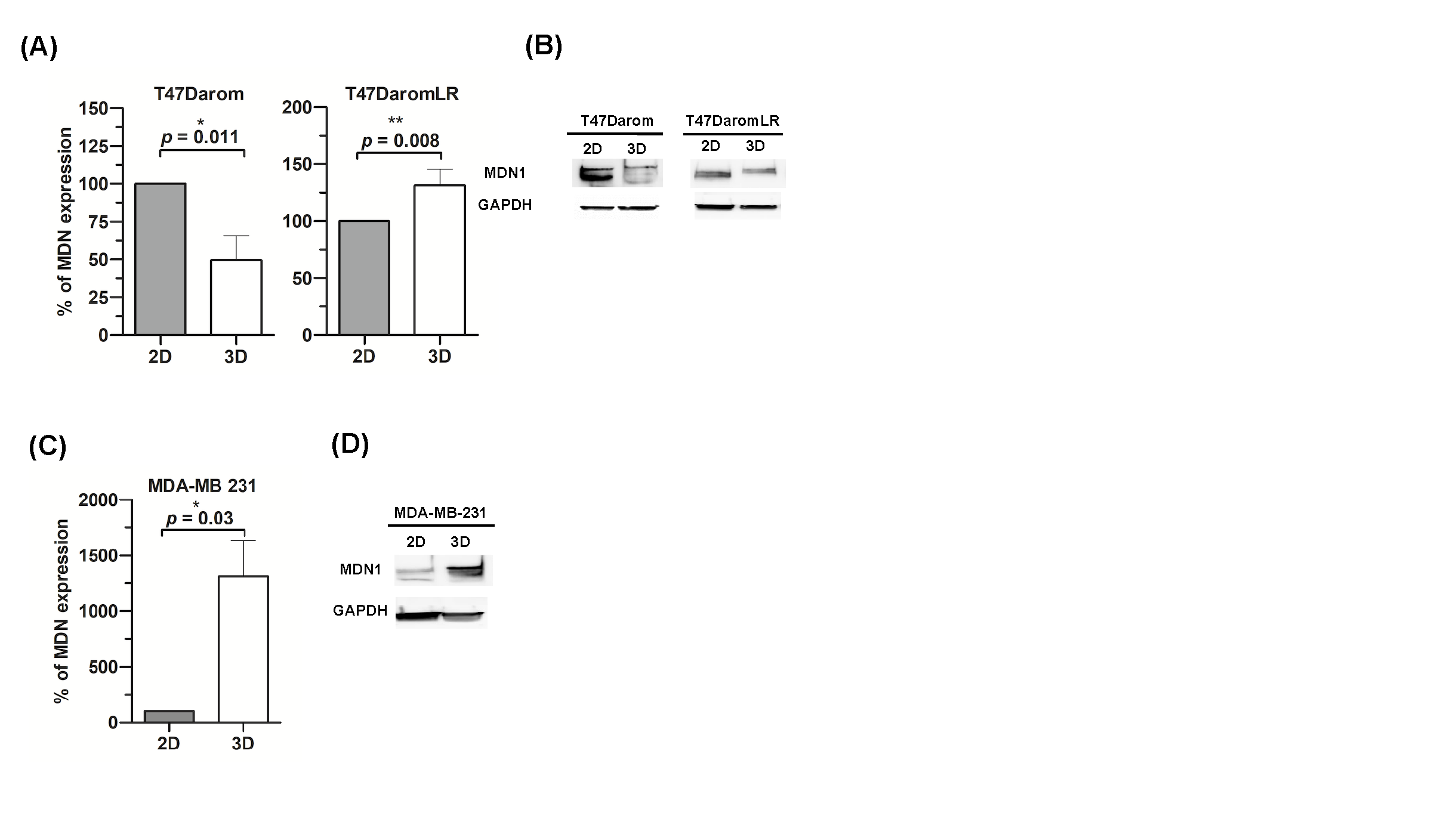

Supplement: Figure S1 — Western Blot analysis of midasin expression. Immunoblots of midasin protein expression in letrozole-sensitive (T47Darom), letrozole-resistant (T47DaromLR) breast cancer cells and MDA-MB 231 triple negative breast cancer cells cultured adherently (2D) or as mammospheres (3D). All cells were evaluated by immunoblot to examine the expression of midasin and GAPDH (loading control). (A, C) Graphs depict normalized percentages of protein expression intensities relative to 2D cell counterparts. (B, D) Representative immunoblot depicts the protein expression of midasin and GAPDH. [file Image_1.tiff]
